# The association between dietary inflammatory index, dietary antioxidant index, and mental health in adolescent girls: an analytical study

**DOI:** 10.1186/s12889-022-13879-2

**Published:** 2022-08-09

**Authors:** Parvin Dehghan, Marzieh Nejati, Farhad Vahid, Amir Almasi-Hashiani, Sevda Saleh-Ghadimi, Reza Parsi, Hamed Jafari-Vayghan, Nitin Shivappa, James R. Hébert

**Affiliations:** 1 Nutrition Research Center, Faculty of Nutrition and Food Sciences, Tabriz University of Medical Sciences, Tabriz, Iran; 2 Department of Biochemistry and Diet Therapy, Faculty of Nutrition and Food Sciences, Tabriz University of Medical Sciences, Tabriz, Iran; 3 Student Research Committee, Tabriz University of Medical Sciences, Tabriz, Iran; 4 Population Health Department, Nutrition and Health Group, Luxembourg Institute of Health, Strassen, Luxembourg; 5 Department of Epidemiology, School of Health, Arak University of Medical Sciences, Arak, Iran; 6 Clinical Research Development Unit of Tabriz Valiasr Hospital, Tabriz University of Medical Sciences, Tabriz, Iran; 7 Department of Nutrition, School of Health, Arak University of Medical Sciences, Arak, Iran; 8 Department of Epidemiology and Biostatistics, Arnold School of Public Health, University of South Carolina, Columbia, SC 29208 USA; 9 Cancer Prevention and Control Program, Arnold School of Public Health, University of South Carolina, Columbia, SC 29208 USA

**Keywords:** Dietary inflammatory index, Dietary antioxidant index, Mental health, Adolescent girls

## Abstract

**Background:**

Diet is considered as one of the modifiable factors that appears to exert a vital role in psychological status. In this way, we designed this study to examine the association between dietary inflammatory index (DII), dietary antioxidant index (DAI), and mental health in female adolescents.

**Methods:**

This cross-sectional study included 364 female adolescents selected from high schools in the five regions of Tabriz, Iran. A 3-day food record was used to extract the dietary data and calculate DII/DAI scores. DII and DAI were estimated to assess the odds of depression, anxiety, and stress based on the Depression Anxiety Stress Scales-21. Adjusted relationships of the DII and DAI with depression, anxiety, and stress were determined using multiple regression after adjusting for age, energy intake, BMI, family income and mother and father education. Overweight was defined as body mass index (BMI)-for-age >  + 1 z-score relative to world health organization standards.

**Results:**

Depression, anxiety, and stress were observed in 21.4%, 26.6%, and 25.7% of subjects, respectively. The percentage of overweight among adolescents was 19.4%. The association between DII and score of mental health profile was positive among subjects in the third tertile of DII compared to subjects in the first tertile. However, this association was not statistically significant after adjusting for confounding variables. Moreover, there was a significant inverse association between DAI and depression and anxiety; and a statistically insignificant association between DAI and stress after adjusting for confounders.

**Conclusions:**

Our results highlighted the importance of a healthy and anti-inflammatory diet on mental health in female adolescents. Therefore, modifying unhealthy dietary habits are likely to be effective in the management of psychosocial disorders.

## Background

Mental disorders can contribute to the higher risk of chronic diseases, years lost due to disability, and mortality among people [1, 2]. Depression and anxiety are two common mental disorders worldwide and are also more common among females than males [3]. According to an Iranian report, females are more likely than males to express mental disorders (28.2% compared to 19.28%) [4]. Iranian studies that examined depression, anxiety, and stress based on the Depression Anxiety Stress Scales-21 item (DASS-21) have shown a consistent result; they reported a higher mean score for all three parameters in females than males [5, 6]. Additionally, it is noted that 10 to 20% of adolescents (aged 10–19 years) are affected by mental disorders, which makes them vulnerable to poor mental health and related physical problems, including infection, respiratory conditions, and weight problems [7, 8]. Overall, mental disorders have been indicated to correspond to 13% of the global burden of disease and injury in adolescents [9]. Considering the high burden of this condition that adversely affects the quality of this critical period of life and its high prevalence among female adolescents, it is crucial to assess the practical approaches that attenuate this disorder.

Social support, socioeconomic status (SES), and health behaviors are affecting factors that concern nutritional status and could influence mental health [10, 11]. Therefore, along with various factors, diet is a critical modifiable factor that appears to have a vital role in psychological status [12]. Studies concerning healthy dietary patterns, nutritional factors, and dietary habits indicate the diets which are high in vegetables, fruits, whole grains, fish, lean meats, and nuts, including the Mediterranean diet, Norwegian diet, and the Prudent diet, are associated with a lower risk of mental disorders [13–16]. While, unhealthy dietary patterns such as a western diet high in red meat, processed products, saturated fat, alcohol, and sugar are linked to a higher risk of mental disorders. These unhealthy dietary patterns are known as pro-inflammatory factors that trigger the induction of inflammation [13–15, 17]. Oxidative stress is induced by inflammation, which lowers cellular antioxidant capacity [18]. Investigations indicate that diets with high antioxidant content may play a key role in modulating inflammation [19]. In the context of the indicated investigations, it seems that some nutritional assessment tools such as dietary inflammatory index (DII) and dietary antioxidant index (DAI) [20, 21] can be used as a practical strategy for assessing the nutritional status and related mental health [22].

The DII has been developed to determine the pro- and anti-inflammatory potential of the whole diet [23] and has been demonstrated to be related to inflammatory biomarkers [24–26]. Several studies have conducted investigations into the relationship of DII and conditions, including metabolic syndrome in American and French adults [27, 28], cardiovascular disease in French and Spanish adults [28, 29], cancer in postmenopausal American women, French, Italian, and American adults [30–36], and mortalities in British adults [37, 38]. Additionally, this index has been validated in Iran [39, 40]. To date, limited studies have examined the relationship between DII and mental health. We are aware of studies concentrating on DII and depression and anxiety [13, 41–45], but little attention has been devoted to DII and other mental health parameters.

The DAI is used to estimate antioxidant content in the whole diet [46, 47]. The relationship between the DAI and the risk of several diseases such as metabolic syndrome [48], cancer [49], cardiovascular disease [50], and although mortality [51, 52] has been shown recently. Studies regarding dietary total antioxidant capacity (DTAC) and mental health parameters, including stress, depression, and anxiety, also indicated that DTAC was inversely associated with these mental health parameters [20, 21, 53–55]. Therefore, due to the association between DTAC and mental health problems, it seems that DAI may be used as a key tool for reducing mental health problems. In the current study, the DAI was used as a comprehensive tool that can evaluate the whole diet, while other related tools like dietary antioxidant quality score can assess only limited micronutrients [56]. In previous studies, the effect of single micronutrients affecting the antioxidant system was mainly investigated [57, 58], but in the DAI, the impact of six major micronutrients with an antioxidant role is examined as an index [59]. Using this index allows researchers to analyze the effects of antioxidants more comprehensively.

As far as we are aware, no previous study has evaluated the association of DII and DAI with depression, anxiety, and stress in female adolescents. Given the limited data, we aimed to assess DII and DAI's association with depression, anxiety, and stress in Iranian adolescent girls.

## Methods

### Study design and setting

This descriptive-analytical study is a part of a larger study to identify the association of nutrient patterns with mental health in Tabriz, Iran. The study population included adolescent girls aged 14 to 16 years selected from high schools in the five regions of Tabriz, Iran. Sampling and data collection were carried out between November 2017 and July 2018.

### Participants and sampling

**Fig. 1:**
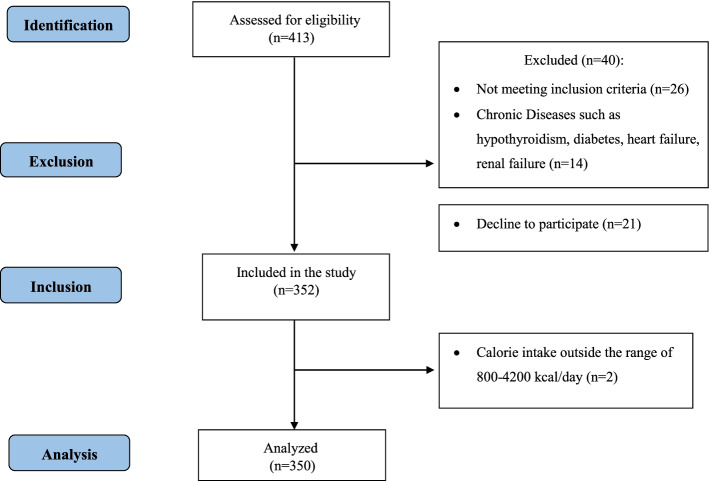
Flowchart of the study

The eligibility to participate in the study were: a) being high school students; b) being female; c) being 14–16 years old. Criteria for exclusion from the study included adherence to special diets, the presence of any apparent clinical illness including endocrine and chronic diseases (thyroid disorders, diabetes, heart, and renal failure) based on the patient's self-reported medical history. Also, subjects with caloric intake outside the range of 800–4200 kcal per day were excluded after collecting dietary intake data [60]. Of all eligible female students in each school, those whose parents did not sign the consent form were classified as “refusal to participate in the study” and were not entered the study. Sampling was done in two stages. In the first stage, schools were selected by cluster sampling from different areas of Tabriz (according to the number of high schools in each urban area and the number of students in these schools). Since SES is related to diet and psychological status, Tabriz city was first divided into three areas (good, moderate, and poor) in terms of SES. In the second stage, 3 schools from good, 3 from poor, and 4 from moderate SES status were selected (the total number of schools at this stage has been 10). The sampling was done in selected schools among girls aged 14 to 16 years. In this stage, 352 students from the selected schools were included based on the eligibility criteria by convenient sampling method (approximately 35 students from each school). Two more subjects were excluded after dietary data collection because their calorie intake was outside the range of 800–4200 kcal/day (Fig. 1). Then, a general questionnaire was completed by interviewing with participants.

### Assessment of anthropometric indices

All anthropometric measurements were performed twice. Then the average of the two measurements was recorded. All participants wore light clothing and no shoes; weight and height were measured using a standardized scale (Seca, Germany) and a portable stadiometer (Seca, Germany). The weight was logged to the nearest 100 g, and height was logged to the closest 0.5 cm. The body-mass index (BMI) was calculated as the ratio of weight in kilograms divided by the square of the height in meters (kg/m^2^). BMI was reported as BMI z-score standardized for 5–19 years old girls [61]. World health organization cut off points were used to define if participants were severe thin (BMI z-score < -3), thin (BMI z-score < -2), had a normal weight (-2 < BMI z-score < 0 and 0 < BMI z-score <  + 1), were overweight (BMI z-score >  + 1) or obese (BMI z-score >  + 2). Two trained nutritionists participated collecting anthropometric measurements.

### Assessment of dietary intake

Participants were asked to record the type and amount of foods and drinks they consumed in a 3-day food record, which is an open dietary report not yes/no questionnaire. They were asked to record on two specific consecutive weekdays and one weekend day. Participants were trained to fill out the food records, and instructions were provided on how to record the quantity using standard household measures. A trained dietitian finally checked all questionnaires in face-to-face interviews. Dietary intake analysis was performed using a food composition table from the last update of U.S. Department of Agriculture website to extract the food related data [62].

### Calculation of dietary inflammatory index (DII)

The 3-day food records were used to extract dietary data and calculate DII scores for all participants. In the food record, the portion size of food items is recorded, and then these data are converted to grams per day of macro and micronutrients. The DII was designed based on the literature published through 2010 and updated in 2014, linking diet to inflammation. Individuals’ intakes of food parameters on which the DII is based are then compared to a world standard database. A complete explanation of the DII is available elsewhere [23]. An explanation of validation work, including DII derived from both dietary recalls and a structured questionnaire similar to an food frequency questionnaire and related to high-sensitivity C-reactive protein interval values, is also available [23]. Briefly, for calculating DII, the dietary data were first linked to the regionally representative world database, which provided a robust estimate of each parameter's mean and standard deviation [23]. These then become the multipliers to express an individual’s exposure relative to the “standard global mean” as a z-score. This is achieved by subtracting the “standard global mean” from the amount reported and dividing this value by the standard deviation. This value is then converted to a centered percentile score to minimize the effect of “right skewing” (a common occurrence with dietary data). The centered percentile score for each food parameter for each individual was then multiplied by the respective food parameter effect score, which is derived from the literature review, to obtain a food parameter-specific DII score for an individual. All of the food parameter-specific DII scores are then summed to create the overall DII score for every participant in the study [23]. DII = b1*n1 + b2*n2………..b31*n31, where b refers to the literature-derived inflammatory effects score for each of the evaluable food parameters and n refers to the food parameter-specific centered percentiles, which were derived from this case–control’s dietary data. Of the theoretically possible list of 45 food parameters, a total of 31 were available from the 3-day food records and therefore could be used to calculate DII (energy, carbohydrate, protein, total fat, fiber, cholesterol, saturated fat, monounsaturated fat, polyunsaturated fat, omega-3, omega-6, niacin, thiamin, riboflavin, vitamin B12, vitamin B6, iron, magnesium, selenium, zinc, vitamin A, vitamin C, vitamin D, vitamin E, folic acid, beta carotene, garlic, ginger, onion, turmeric, saffron, pepper).

### Calculation of dietary antioxidant index (DAI)

The DAI for all participants was calculated based on 3-day food records data. For estimating the DAI, each of the same six dietary vitamins and minerals was standardized by subtracting the global mean and dividing the result by the global standard deviation. The calculation of the DAI was done by summing up the standardized intakes of these vitamins and minerals and equal weight, as follows [47]:

$$\mathrm{DAI}=\sum_{i=1}^{\mathrm n=6}.\frac{\mathrm{Individual}\;\mathrm{Intake}-\mathrm{Mean}}{\mathrm{SD}}$$

### Assessment of mental health profile

The Depression, Anxiety, and Stress scale (DASS) is a validated and reliable questionnaire for assessing psychological disorders [63]. The DASS is a modified version of the Depression Anxiety Stress Scales-42 [63]. The validity and reliability of the questionnaire have been established in a sample of Iranian population with acceptable internal consistency (*α* = 0.84 to 0.91) and satisfactory convergent validity [64]. The internal consistency of the Persian DASS-21 was also very good in an adolescent sample (*α* = 0.86) [65].

The DASS consists of 21 items and three subscales for anxiety, depression, and stress, seven items in each category. There is a 4-point scale from 0 to 3 for scoring the items; the 0 is “did not apply to me at all,” and three is “applied to me very much or most of the time.” Item scores are summed for each of the seven-item subscales, ranging from 0 to 21 for each subscale and a total possible score of 63 for the entire scale. Lovibond and Lovibond’s [66] cut-off values were used to rate the severity of each of the outcomes which are as follows:

- Severity of depression: 0–9 (normal), 10–13 (mild),14–20 (moderate), 21–27 (severe), 28^+^ (extremely severe).
- Severity of anxiety: 0–7 (normal), 8–9 (mild), 10–14 (moderate), 15–19 (severe), 20^+^ (extremely severe).
- Severity of stress: 0–14 (normal), 15–18 (mild),19–25 (moderate), 26–33 (severe), 34^+^ (extremely severe).

### Sample size

At the time of designing the study, based on our search, no similar study was found, so the correlation formula was used to determine the sample size. The correlation coefficient between the score of nutrient intake pattern and depression was about 0.15 based on a pilot study. The first type of error was 5%, the study power was 80%, and using the following formula, the sample size was estimated to be 347 people. Increasing to 360 people with anticipation of an overall dropout:

- The standard normal deviate for α = Z_α_ = 1.9600.
- The standard normal deviate for β = Z_β_ = 0.8416.
- C = 0.5 * ln[(1 + r)/(1-r)] = 0.1511.
- Total sample size = N = [(Z_α_ + Z_β_)/C]^2^ + 3 = 347.

### Statistical analysis

Normally-distributed continuous data are presented as mean and standard deviation (SD); qualitative data are presented as frequency (percent). The Kolmogorov–Smirnov test was used to determine the normality of distribution for continuous variables. One-way analysis of variance (ANOVA) was applied to compare continuous demographic variables. Categorical variables were compared using the chi-square test across tertiles of DII. To examine the mean differences of nutrient intakes across tertiles of DII, One-way ANOVA and Kruskal–Wallis H test were used in normal and non-normal distributed variables, respectively.

Univariate linear regression was conducted to determine the association between DII and DAI with depression, anxiety, and stress. The relationships of the DII and DAI with depression, anxiety, and stress were determined using generalized linear model (GLM) adjusted for confounders in 3 models. The models were defined as follows; model 1: crude, model 2: Adjusted for age and BMI, Model 3: Model 2 + Adjusted for energy intake, family income and mother and father education. The GLM with Gaussian family and identity link were used. Regarding confounding variables, based on prior knowledge and review of articles [23, 40, 59, 67], variables that were the common cause of exposure (DII, DAI) and outcome (Mental health profile) were selected as confounding variables, and their role was adjusted in the analysis. For example, family income is related to both DII, DAI, and mental health. Therefore, it can be considered as a confounding variable in the assessment of this relationship. *P* < 0.05 was considered statistically significant. Data analyses were performed using SPSS 26 (SPSS Inc., Chicago, IL, USA).

## Results

The study flowchart was depicted in Fig. 1. The demographic findings of the participants are presented in Table 1 based on DII and DAI tertiles. The mean (SD) of age for all participants was 15.4 (1.1). The frequency of normal weight and overweight based on BMI z-score for all participants was 69.8%, 19.4%, respectively. Depression, anxiety, and stress were present in 21.4%, 26.6%, and 25.7% of the subjects, respectively. Demographic variables including mother and father education and father job were significantly different between DII tertiles (*p* < 0.05). However, there was no statistically significant difference considering other demographic variables including weight, height, BMI z-scores, mother job and family income. In different tertiles of DAI, the mean (SD) of age was significantly different (*p* = 0.009). Other demographic variables were not statistically different.

**Table 1:** Demographic characteristics of the study subjects (*n* = 350)

Variable	Total (*n* = 350)	Dietary Inflammatory Index (DII)				Dietary Antioxidant Index (DAI)			
		**Tertile 1 (< 2.31) (*n* = 119)**	**Tertile 2 (2.31 to 3.42) (*n* = 114)**	**Tertile 3 (> 3.42) (*n* = 117)**	***P***	**Tertile 1 (< -2.00) (*n* = 117)**	**Tertile 2 (-2.00 to 0.74) (*n* = 116)**	**Tertile 3 (> 0.74) (*n* = 117)**	***P***
**Age (years)**	15.4 (1.1)	15.5 (1.1)	15.4 (1.2)	15.4 (1.1)	0.894	15.7 (1.2)	15.4 (1.1)	15.2 (1.1)	0.009
**Weight (kg)**	57.2 (11.9)	55.7 (11.0)	57.9 (12.4)	58.1 (12.2)	0.207	58.6 (13.0)	57.1 (11.6)	56.0 (10.9)	0.228
**Height (cm)**	161.1 (5.58)	160.7 (5.7)	161.7 (5.6)	160.9 (5.4)	0.362	160.8 (5.3)	161.6 (5.8)	161.1 (5.6)	0.621
**BMI z-score**									
Severe Thin	7 (2.1)	4 (3.5)	1 (0.9)	2 (1.8)	0.249	2 (1.7)	1 (0.9)	4 (3.4)	0.518
Thin	30 (8.8)	13 (11.3)	13 (11.6)	4 (3.5)		6 (5.2)	11 (10.1)	13 (11.2)	
Normal	238 (69.8)	76 (66.1)	76 (67.9)	86 (75.4)		82 (70.7)	77 (70.6)	79 (68.1)	
Overweight	66 (19.4)	22 (19.1)	22(19.7)	22 (19.3)		26 (22.5)	20 (18.3)	20 (17.2)	
**Mother education**									
Illiterate	11 (3.17)	4 (3.4)	4 (3.6)	3 (2.6)	0.012	5 (4.3)	3 (2.6)	3 (2.6)	0.365
Under Diploma	149 (43.0)	34 (28.8)	53 (47.3)	62 (53.0)		48 (41.0)	51 (45.1)	50 (42.7)	
Diploma	130 (37.5)	56 (47.5)	36 (32.1)	38 (32.5)		39 (33.3)	40 (35.4)	51 (43.6)	
Academic	57 (16.4)	24 (20.3)	19 (17.0)	14 (12.0)		25 (21.4)	19 (16.8)	13 (11.1)	
**Father Education**									
Illiterate	8 (2.33)	3 (2.6)	3 (2.7)	2 (1.7)	0.001	5 (4.3)	2 (1.7)	1 (0.88)	0.367
Under Diploma	138 (40.1)	29 (24.8)	51 (45.9)	58 (50.0)		45 (38.8)	53 (46.1)	40 (35.4)	
Diploma	104 (30.2)	51 (43.6)	26 (23.4)	27 (23.3)		34 (29.3)	30 (26.1)	40 (35.4)	
Academic	94 (27.3)	34 (29.1)	31 (27.9)	29 (25.0)		32 (27.6)	30 (26.1)	32 (28.3)	
**Mother Job**									
Housewife	305 (89.2)	99 (85.3)	101 (89.4)	105 (92.9)	0.332	102 (88.7)	100 (88.5)	103 (90.3)	0.973
Retired	7 (2.1)	2 (1.7)	3 (2.6)	2 (1.8)		3 (2.6)	2 (1.8)	2 (1.7)	
Employed	30 (8.8)	15 (12.9)	9 (8.0)	6 (5.3)		10 (8.7)	11 (9.7)	9 (7.9)	
**Father Job**									
Retired	39 (12.0)	16 (14.2)	15 (14.4)	8 (7.4)	0.029	15 (14.0)	10 (9.1)	14 (13.0)	0.732
Unemployed	187 (57.5)	55 (48.7)	57 (54.8)	75 (69.4)		63 (58.9)	64 (58.2)	60 (55.6)	
Employed	99 (30.5)	42 (37.2)	32 (30.8)	25 (23.1)		29 (27.1)	36 (32.7)	34 (31.5)	
**Family Income**									
Low	41 (12.3)	15 (13.2)	17 (15.6)	9 (8.2)	0.169	18 (16.2)	12 (10.7)	11 (10.0)	0.632
Moderate	260 (78.1)	84 (73.7)	82 (75.2)	94 (85.4)		84 (75.7)	88 (78.6)	88 (80.0)	
High	32 (9.6)	15 (13.2)	10 (9.2)	7 (6.4)		9 (8.1)	12 (10.7)	11 (10.0)	

Continuous variables are expressed as mean (SD); categorical variables are expressed as count (percentages). One-Way ANOVA is used for continuous variables and Chi-Square test is used for categorical variables

*BMI* Body mass index

Distribution of energy and nutrient intake according to the DII and DAI tertiles is shown in Table 2. Significant differences in energy and nutrients were observed in the DAI tertiles but did not differ among the DII tertiles. Linear regression analysis of the association between DII and score of depression, anxiety, and stress showed that this association was not statistically significant after adjusting for confounding variables including: age, energy intake, BMI, family income and mother and father education (Table 3).

**Table 2:** Dietary intakes of subjects across tertiles of dietary inflammatory index (DII) and dietary antioxidant index (DAI) ^a^

Variable	Dietary Inflammatory Index (DII)				Dietary Antioxidant Index (DAI)			
	**Tertile 1 (< 2.31) (*n* = 119)**	**Tertile 2 (2.31 to 3.42) (*n* = 114)**	**Tertile 3 (> 3.42) (*n* = 117)**	***P***	**Tertile 1 (< -2.00) (*n* = 117)**	**Tertile 2 (-2.00 to 0.74) (*n* = 116)**	**Tertile 3 (> 0.74) (*n* = 117)**	***P***
**Energy (kcal/day) **^**b**^	1889.07 (899.67)	1819.95 (885.31)	1940.72 (999.66)	0.613	1327.49 (460.70)	1709.97 (527.44)	2612.52 (1107.91)	** < 0.001**
**Carbohydrate (g/day) **^**b**^	286.53 (118.01)	278.48 (110.08)	283.05 (123.21)	0.871	217.90 (82.49)	263.29 (76.35)	366.88 (129.76)	** < 0.001**
**Protein (g/day) **^**b**^	66.44 (27.80)	63.32 (26.61)	64.29 (29.75)	0.625	49.07 (14.63)	56.70 (16.46)	87.05 (36.95)	** < 0.001**
**Fat (g/day)**	46.66 (30.88–75.00)	48.09 (29.00–64.12)	49.00 (33.95–73.14)	0.341	30.08 (21.72–40.97)	48.09 (34.00–70.00)	71.32 (55.04–98.33)	** < 0.001**
**SFA (g/day)**	16.30 (10.99–23.67)	15.80 (10.90–21.95)	16.36 (12.42–25.50)	0.223	11.30 (8.39–14.33)	16.15 (12.24–21.95)	23.00 (18.55–34.45)	** < 0.001**
**MUFA (g/day)**	13.00 (8.94–21.98)	14.32 (7.58–19.47)	15.00 (9.31–21.36)	0.303	8.73 (5.32–12.14)	14.03 (9.57–20.42)	21.66 (16.65–28.80)	** < 0.001**
**PUFA (g/day)**	10.58 (5.22–16.70)	10.67 (4.33–17.49)	9.54 (5.94–16.45)	0.968	5.70 (2.84–8.99)	10.04 (5.45–16.50)	16.66 (11.90–26.90)	** < 0.001**
**Linoleic Acids (g/day)**	9.03 (4.49–15.30)	9.45 (3.68–16.06)	8.68 (4.60–15.30)	0.970	4.29 (1.94–7.55)	9.03 (4.37–15.67)	15.00 (10.38–23.94)	** < 0.001**
**Linolenic Acids (g/day)**	0.19 (0.06–0.38)	0.13 (0.05–0.40)	0.11 (0.45–0.34)	0.650	0.06 (0.01–0.12)	0.18 (0.05–0.37)	0.30 (0.13–0.57)	** < 0.001**
**Dietary Fiber (g/day) **^**b**^	13.31 (7.07)	11.82 (5.03)	13.44 (7.35)	0.117	8.25 (4.04)	12.80 (5.46)	17.54 (6.45)	** < 0.001**
**Vitamin A (RE/day)**	658.90 (394.00–1081.00)	671.45 (342.25–1014.25)	593.10 (343.55–1033.50)	0.850	378.50 (228.70–555.35)	659.45 (388.52–933.25)	1038.00 (672.00–1888.00)	** < 0.001**
**Vitamin D (µg/day)**	0.90 (0.12–1.97)	0.81 (0.06–2.09)	0.52 (0.04–1.81)	0.787	0.18 (0.02–1.27)	0.82 (0.10–2.09)	1.16 (0.35–2.40)	** < 0.001**
**Vitamin K (µg/day)**	43.20 (22.37–90.54)	39.65 (19.60–67.70)	37.00 (17.94–62.68)	0.157	22.21 (13.95–39.24)	42.68 (20.62–75.95)	61.80 (35.67–144.90)	** < 0.001**
**α-Tocopherol (mg/day)**	4.43 (2.07–8.41)	4.11 (2.45–7.18)	3.70 (2.53–6.22)	0.965	2.51 (1.68–3.71)	4.00 (2.47–6.33)	7.38 (4.30–12.42)	** < 0.001**
**Vitamin C (mg/day)**	68.27 (39.50–115.00)	61.77 (39.32–105.20)	64.86 (41.62–104.95)	0.775	36.30 (18.41–49.08)	73.30 (49.34–96.22)	118.20 (84.62–156.00)	** < 0.001**
**Calcium (mg/day) **^**b**^	580.51 (278.05)	573.04 (321.58)	533.38 (287.90)	0.425	436.67 (182.51)	523.37 (230.91)	726.60 (364.32)	** < 0.001**
**Iron (mg/day) **^**b**^	13.80 (6.46)	12.76 (5.22)	13.86 (7.38)	0.342	9.31 (2.70)	12.57 (3.88)	18.56 (7.59)	** < 0.001**
**Zinc (mg/day)**	6.14 (4.91–8.71)	6.05 (4.64–7.31)	6.00 (4.96–7.93)	0.495	4.60 (3.71–5.38)	5.93 (5.06–6.91)	8.90 (7.12–11.24)	** < 0.001**
**Copper (mg/day)**	0.91 (0.65–1.30)	0.88 (0.62–1.25)	0.93 (0.63–1.23)	0.679	0.62 (0.46–0.79)	0.90 (0.70–1.19)	1.25 (0.99–1.87)	** < 0.001**
**Selenium (mg/day)**	0.05 (0.03–0.09)	0.05 (0.02–0.08)	0.05 (0.03–0.07)	0.728	0.03 (0.01–0.05)	0.05 (0.03–0.07)	0.08 (0.05–0.12)	** < 0.001**

*SFA* Saturated fatty acid, *MUFA* Mono unsaturated fatty acid, *PUFA* Poly unsaturated fatty acid

^a^Values are expressed as median (25th–75th percentile) and P-value based on Kruskal–Wallis H test

^b^Values are expressed as mean (SD) and *P*-value based on One-Way ANOVA

**Table 3:** Association of dietary inflammatory index and mental health disorders profile’s scores

Mental health profile	B (95%CI)			
	**Tertile 1 (< 2.31) (*n* = 119)**	**Tertile 2 (2.31 to 3.42) (*n* = 114)**	**Tertile 3 (> 3.42) (*n* = 117)**	***P* trend**
***Depression***				
Model 1	Ref	-0.002 (-0.96 to -0.96)	0.58 (-0.37 to 1.54)	0.230
Model 2	Ref	-0.15 (-1.12 to 0.83)	0.45 (-0.52 to 1.43)	0.359
Model 3	Ref	-0.27 (-1.29 to 0.75)	0.32 (-0.69 to 1.34)	0.536
***Anxiety***				
Model 1	Ref	-1.05 (-2.31 to 0.20)	0.37 (-0.87 to 1.62)	0.566
Model 2	Ref	-1.25 (-2.51 to 0.004)	0.29 (-0.95 to 1.55)	0.645
Model 3	Ref	-1.20 (-2.50 to -0.09)	0.46 (-0.84 to 1.76)	0.488
***Stress***				
Model 1	Ref	-0.35 (-1.46 to 0.74)	0.11 (-0.97 to 1.21)	0.836
Model 2	Ref	-0.54 (-1.66 to 0.57)	-0.0001 (-1.11 to 1.11)	0.999
Model 3	Ref	-0.57 (-1.73 to 0.59)	0.14 (-1.02 to 1.31)	0.806

**Model 1:** Crude, **Model 2:** Adjusted for age and BMI, **Model 3:** Model 2 + Adjusted for energy intake, family income and mother and father education

*P* for trend based on linear regression analysis

Table 4 represents the data of association between DAI and score of mental health profile. After adjusting for confounders, there was a significant inverse association of DAI with depression and anxiety. However, there was not a statistically significant association between DAI and stress.

**Table 4:** Association of dietary antioxidant index and mental health disorders profile’s scores

Mental health profile	B (95%CI)			
	**Tertile 1 (< -2.00) (*n* = 117)**	**Tertile 2 (-2.00 to 0.74) (*n* = 116)**	**Tertile 3 (> 0.74) (*n* = 117)**	***P* trend**
***Depression***				
Model 1	Ref	-1.17 (-2.13 to -0.21)	-1.25 (-2.21 to -0.30)	**0.010**
Model 2	Ref	-0.96 (-1.94 to 0.02)	-1.20 (-2.17 to -0.22)	**0.016**
Model 3	Ref	-1.006 (-2.04 to 0.02)	-1.29 (-2.53 to -0.04)	**0.034**
***Anxiety***				
Model 1	Ref	-1.65 (-2.90 to -0.40)	-1.79 (-3.03 to -0.54)	**0.005**
Model 2	Ref	-1.30 (-2.57 to -0.03)	-1.51 (-2.78 to -0.25)	**0.019**
Model 3	Ref	-1.22 (-2.55 to 0.10)	-1.60 (-3.21 to -0.003)	**0.041**
***Stress***				
Model 1	Ref	-0.69 (-1.78 to -0.40)	-1.11 (-2.20 to -0.01)	**0.046**
Model 2	Ref	-0.56 (-1.69 to 0.56)	-0.98 (-2.10 to 0.14)	0.087
Model 3	Ref	-0.52 (-1.71 to 0.66)	-0.98 (-2.42 to 0.45)	0.175

**Model 1:** Crude, **Model 2:** Adjusted for age and BMI, **Model 3:** Model 2 + Adjusted for energy intake, family income and mother and father education

*P* for trend based on linear regression analysis

## Discussion

The present analytical study examining the association of DII and DAI with depression, anxiety, and stress in Iranian females of 14 to 16 years old, revealed a significant negative association between DAI and depression, anxiety, and stress. Besides, the results demonstrate a non-significant positive association between DII and mental health profile score. To date, this study is the first which simultaneously investigates the association of DAI and DII with depression, anxiety, and stress in female adolescents.

We found that there is a non-significant positive association between DII and depression, anxiety, and stress. This finding agrees with a recent study conducted on 3523 participants from France, aged 35–60 years, who were initially free of depressive symptoms. The current prospective study reported no remarkable relationship between DII and depressive symptoms among women. However, a marginally significant link was seen among men [68]. Moreover, another study regarding DII and anxiety among 11,592 United States adults > 20 years indicated no association between the mentioned parameters [69]. Our results were in line with another cross-sectional study carried out on 7083 adults aged 35 to 65 years in Iran. The mentioned study did not report a significant association between DII and depression among men. However, a remarkable association was reported among women [43]. Our findings contrast with other studies conducted on Iranian adolescent girls, which indicated that a higher DII was significantly associated with higher odds of depression and stress levels in adolescent girls of Tehran [70, 71]; Therefore, due to the different study regions, factors such as residence, ethnicity, local eating habits, would explain these differences. Also, Sánchez-Villegas et al*.* assessed 15,093 Spanish participants in a cohort study and found that a higher DII was associated with a higher risk of depression. Furthermore, they reported that this correlation was stronger among older individuals and others with cardiometabolic comorbidities [45]. The result’s inconsistency may be due to differences in sample size, study populations, geographic areas, study design, eating behavior questionnaires, cooking methods, and applied indices. Regarding statistical procedures, it should be mentioned that Villegas et al*.* conducted a cohort study and calculated adjusted hazard ratio using the Cox method, while in our study, due to the cross-sectional design of it, linear regression method was used for analysis and adjusted regression coefficient has been reported in the current study. Therefore, the different statistical procedures could be a reason for the discrepancy between the findings.

Although the relationship between DII and assessed parameters was insignificant, the positive reported association could be considered clinically noteworthy. The mechanisms through which the higher DII scores might induce mental disorders are not entirely elucidated. However, the presented mechanisms propose that higher DII increases the level of inflammatory biomarkers, which may interact with neural function. The released cytokines such as interleukin **(**IL)-6, IL-1Ɓ, and tumour necrosis factor alpha develop depression by changing the metabolism of the neurotransmitters [72–74]. Another proposed route is concentrating on the inflammation, stress, and hypothalamic–pituitary–adrenal axis. The related research has shown that the higher DII of diet increases the susceptibility to stress which its mechanisms are not entirely clarified [75]. Stress affects the hypothalamic–pituitary–adrenal axis and alters the balance of related neurochemicals, leading to depression [76, 77]. Our results’ non-significant p-value could be due to our sample size, which probably reflects significant results in larger sample sizes.

The DAI significant inverse association with depression and anxiety was noted in the adjusted and unadjusted models. However, the significant relationship between DAI and stress was only observed in the unadjusted model. Our study was in line with the previous findings, which indicated that dietary patterns, which are higher in vegetables, fruits, and fish, demonstrate an inverse relationship with depression [78–80], dysthymia, and anxiety [80]. In addition, other studies reported that lower antioxidant intake in the diet is associated with depression, which does not always appear with a meaningful difference in the antioxidant status of normal and depressed cases [81]. In contrast with present findings, several studies did not report a remarkable relationship between dietary antioxidant capacity and depression [53], anxiety [82], and stress [20].

Based on the existing research, the oxidant-antioxidant imbalance has a crucial role in developing mental disorders. It has been indicated that the high levels of reactive oxygen and nitrogen species may result in the dysfunction of biomolecules such as DNA and mitochondria, which is the underlying cause of the psychiatric disorder [83]. Oxidative stress and DNA damages are explained in light of telomeres. Telomeres are structures that consist of repetitive DNA sequences and are aimed to protect the chromosome ends. The process of telomere shortening leads to DNA damage. High levels of oxidative stress accelerate the telomere shortening, contributing to mental health problems [84, 85]. On the other hand, alternations in the oxidation rate of synaptic molecules and increased oxygen levels result in the decline of neurotransmitters, which play an essential role in increasing the odds of mental health conditions [86, 87].

As the strengths of the current study, we should mention that the use of dietary record in this study reduced the likelihood of recall bias. In addition, the information was collected by a trained expert, which minimized the measurement error. Moreover, the validated questionnaires were used, and a broad range of confounders was controlled. Also, it is noteworthy that DAI and DII have been validated in Iran [40, 59, 67]. Finally, the data were finalized by a nutrition epidemiologist, and its quality was confirmed. However, our findings should be noted in light of potential limitations. The cross-sectional design of the present study cannot determine causality. Also, a validated 3-day food record was used to estimate dietary intake; thus, some measurement errors should be considered. It is also worth noting that there are not any measurements of inflammatory biomarkers in this study. In addition, no data is presented regarding the age of menarche. The present study was carried out on female cases that indicate that future prospective studies should be conducted on both sexes and different study populations with various dietary patterns. Additionally, it is recommended that future studies measure inflammatory biomarkers and provide data regarding the age of menarche to shed light on these points.

## Conclusions

Our findings revealed a significant inverse association between DAI and depression, anxiety, and stress in Iranian females of 14 to 16 years old. Also, a non-significant direct association was observed between DII and related parameters. In addition to the key role of social determinants of health that affects both nutritional status and mental health, concentrating on the diet and modifying the incorrect habits are likely to be effective due to the notable impact of dietary nutrients on mental health.

## Data Availability

The dataset is available from the corresponding author on reasonable request.

## Abbreviations

ANOVA: One-way Analysis of Variance
BMI: Body Mass Index
DAI: Dietary Antioxidant Index
DASS-21: Depression Anxiety Stress Scales-21 item
DII: Dietary Inflammatory Index
DTAC: Dietary Total Antioxidant Capacity
GLM: Generalized Linear Model
IL: Interleukin
SD: Standard Deviation
SES: Socioeconomic Status
